# Correlation between microbiota and growth in Mangrove Killifish (*Kryptolebias marmoratus*) and Atlantic cod (*Gadus morhua*)

**DOI:** 10.1038/srep21192

**Published:** 2016-02-15

**Authors:** Torunn Forberg, Eli Bjørnø Sjulstad, Ingrid Bakke, Yngvar Olsen, Atsushi Hagiwara, Yoshitaka Sakakura, Olav Vadstein

**Affiliations:** 1 Norwegian University of Science and Technology, Department of Biotechnology, N7491 Trondheim, Norway; 2 Norwegian University of Science and Technology, Department of Biology, N7491 Trondheim, Norway; 3 Nagasaki University, Faculty of Fisheries, Division of Marine Biology and Dynamics, 1-14 Bunkyo-machi Nagasaki-shi, 852-8521 Japan; 4 Nagasaki University, Faculty of Fisheries, Division of Marine Life Science and Biochemistry, 1-14 Bunkyo-machi Nagasaki-shi, 852-8521 Japan

## Abstract

The vertebrate gut is host to large communities of bacteria, and one of the beneficial contributions of this commensal gut microbiota is the increased nutritional gain from feed components that the host cannot degrade on its own. Fish larvae of similar age and under the same rearing conditions often diverge with regards to growth. The underlying reasons for this could be differences in genetic background, feeding behavior or digestive capacity. Both feeding behavior and digestion can be influenced by differences in the microbiota. To investigate possible correlations between the size of fish larvae and their gut microbiota, we analyzed the microbiota small and large genetically homogenous killifish and genetically heterogeneous cod larvae by Bray-Curtis Similarity measures of 16S DNA DGGE patterns. A significant difference in richness (p = 0.037) was observed in the gut microbiota of small and large killifish, but the overall gut microbiota was not found to be significantly different (p = 0.13), indicating strong genetic host selection on microbiota composition at the time of sampling. The microbiota of small and large cod larvae was significantly different with regards to evenness and diversity (p = 0.0001), and a strong correlation between microbiota and growth was observed.

The vertebrate gut contains numerous bacteria interacting with each other as well as with their host. The commensal gut microbiota contributes to the “energy harvest” from the diet by breaking down complex carbohydrates into monomers for further microbial fermentation. The end products of this fermentation process, short chain fatty acids (SCFA), are released into the gut and adsorbed by host cells^1^,^2^,^3^. Dietary carbohydrates are less nutritionally significant for carnivorous/planktivorous fish such as cod and killifish, where the main energy yield comes from ingested fats and protein^4^,^5^. However, the gut microbiota may also have an effect on digestion of lipids and fat, as germ-free zebrafish show reduced differentiation of gut epithelia and reduced lipid uptake compared to conventional controls^6^,^7^.

In accordance with the idea of microbes increasing the yield of ingested food, specific members of gut microbiota appear to be positively correlated with obesity in humans and mice^2^,^8^. Obese mice and humans have altered ratios of bacteria belonging to Firmicutes/Bacteroidetes (F/B) compared to their lean counterparts, as well as lower microbial diversity. In a recent paper by Zhao *et al.*^9^, the F/B ratio and causality of obesity is reviewed. Zhao suggests that the F/B ratio is too simple, and that a closer look at the underlying classes of bacteria such as the Mollicutes may be more relevant for how bacteria may contribute to obesity. In addition, Proteobacteria, specifically *Enterobacter cloaca* has been shown to be a co-factor in inducing obesity in gnotobiotic mice, by inducing a low grade inflammatory response that alters the lipid metabolism of the host^10^. Recently, expression of bacterial bile salt hydrolase has been found to direct host gene-expression and influence weight gain in mice^11^.

Fish larvae of similar age and under the same rearing conditions often diverge with regards to size, reflecting differences in growth rate. The underlying reasons for this could be genetic differences, different feeding behavior or digestive capacity. Both feeding behavior and digestive capacity could be influenced by differences in microbiota^12^,^13^. Genetic differences leading to different growth rates may also be indirectly linked to altered microbiota, as recent studies on mice and stickleback (*Gasterosteus aculeatus*) has revealed host QTLs correlated with specific members of the gut microbiota^14^,^15^,^16^. While obesity is considered unwanted in humans, a commensal microbiota that could increase the energy harvest from of feed would be considered a big advantage in farmed animals –including fish. Fish larvae are actively growing, and enhanced energy provision from the feed due to the activity of the gut microbiota would influence the growth rate of the larvae positively. While several probiotic trials claim that addition of specific bacteria improves growth^17^,^18^,^19^,^20^, there is to our knowledge only one published study that investigates gut microbiota and growth rate in fish^21^. However, this study was based on culture-dependent characterization of microbiota.

The objective of this study was to investigate potential correlations between growth and microbiota composition in fish larvae, and to investigate the effects of genetic background on microbiota composition. The microbiota of genetically homogenous Mangrove killifish (*Kryptolebias marmoratus,* clonal lineage DAN) juveniles and genetically heterogeneous Atlantic cod larvae (*Gadus morhua* L.) was characterized by 16S DNA denaturing gel electrophoresis (DGGE). To our knowledge this is the first study investigating correlations between microbiota and growth in fish by the use of culture-independent characterization techniques.

## Results and Discussion

### Growth of DAN killifish

**Figure 1:**
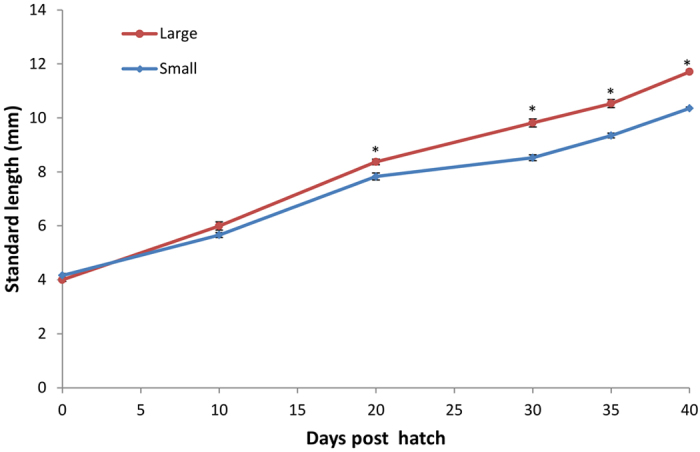
Average growth (standard length in mm) of the 7 smallest and 7 largest DAN killifish larvae, from hatching to the juvenile stage (40 dph). * Statistically significant differences in growth (P < 0.05) from day 20 onwards. Error bars = Standard error.

Fourteen out of the 30 sampled killifish were selected for further analysis based on growth measurements. These two groups consisted of the 7 smallest and 7 largest killifish, referred to as S1-S7 and L1-L7, respectively. The regular length measurements of killifish individuals (Fig. 1) show a separation between the large and small group from about day 10 onwards (P = 0.09), with statistically significant differences from day 20 post hatch (dph) (p < 0.007). After 3 weeks the growth rate slowed down both for the large and small killifish. This is in accordance with previous observations of killifish development^22^,^23^ and coincides with the transition into the juvenile stage. The large differences in length and wet weight of the juveniles suggest a possible influence of microbiota on growth, as both the genetic background and rearing conditions of the killifish were uniform.

**Figure 2:**
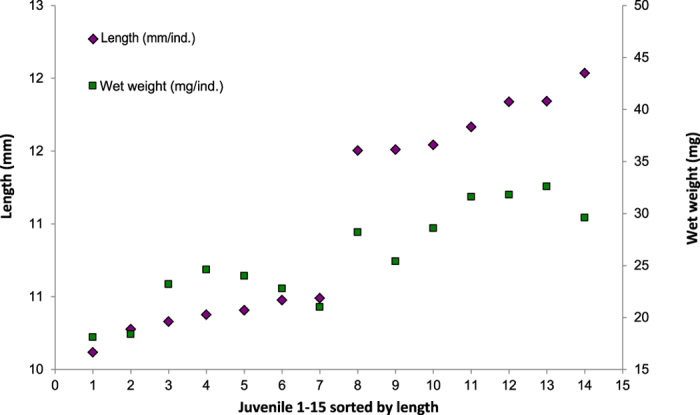
Length and wet weight of the 7 largest and 7 smallest DAN killifish juveniles at 40 dph.

At sampling (40 dph) both the average length and weight of the small juveniles was significantly lower (p < 0.005) than for the large juveniles (Fig. 2) (for individual measurements see Supplementary table S1).

### Microbiota associated with large and small mangrove DAN killifish juveniles

**Figure 3:**
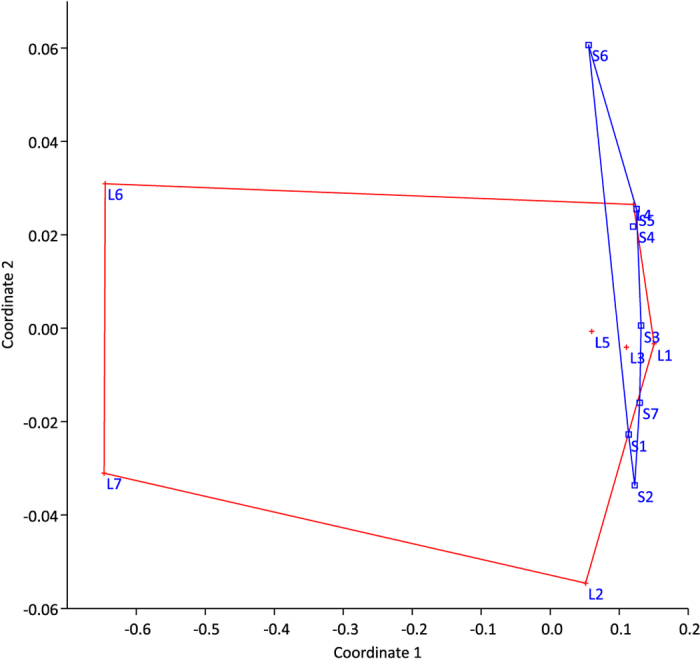
NMDS ordination of GI microbial community profiles using NMDS with Bray-Curtis similarities for large (L1-L7, designated with red crosses) and small (S1–S7, designated with blue squares) DAN killifish juveniles (stress value < 0.2).

Forty-eight unique DGGE bands were detected, and the richness (k) varied between 23 and 34 bands among individuals (for gel image see supplementary Fig. S1). The average k was significantly lower for large killifish juveniles (p = 0.037). Neither the Shannon diversity index (H′) nor the Pielou’s evenness index (J′) showed significant differences (p > 0.05), between large and small killifish. The exploratory non metric multidimensional scaling (NMDS) of DGGE results showed a clear overlap in gut microbiota composition for most of the small and large killifish (Fig. 3). The DGGE profiles of three of the large killifish juveniles form a separate cluster on the NMDS, among these are the two largest individuals (L6 and L7). Hypothesis testing with One-Way PERMANOVA confirmed that there were no significant differences in the gastrointestinal microbiota between the two groups (p = 0.124). Average Bray Curtis similarities were calculated to compare fish, water and *Artemia* samples. Highest similarity (0.53 ± 0.02) was observed when comparing large and small killifish, whereas both water and *Artemia* samples showed low similarity to the killifish (average of 0.33 between killifish and water, and 0.32 between killifish and *Artemia*, see supplementary table S2 for all values and standard errors). Hypothesis testing with One-Way PERMANOVA confirmed that the *Artemia* microbiota was significantly different from the microbiota of both large and small killifish (p = 0.0031 and p = 0.0320, respectively).

As some separation in gut microbiota composition is suggested in the ordination in Fig. 3, we excluded the intermediate size fish (the largest of the small and the smallest of the large) from statistical analysis. One-way PERMANOVA confirmed significant differences in gut microbiota composition when analyzing only fish S1–S5 and L4–L7, P = 0.024.

SIMPER analysis showed subtle differences in abundance of 48 bands from the small and large killifish microbiota. Six bands from the DGGE gel were re-amplified and sequenced, among these were the top three bands identified in the SIMPER analysis. None of the sequenced bands were unique for small or large killifish, but were found in different amounts (Table 1). The Sequenced bands contribute to 27.7% of the variation between the two groups. Two opportunistic *Vibrio* species were among the identified bacteria. However, as there was no sign of infection in the sampled fish which exhibited normal growth and survival, and the highest abundance was found in the large fish, these bacteria were probably not harmful. The effects of the identified bacteria on growth were probably negligible, as the amounts found in the large and small killifish were not significantly different.

Statistically significant differences in gut microbiota composition between small and large killifish was only apparent after excluding the intermediate-size fish from the analysis, with this in mind follow-up studies should avoid sampling continuous sizes of fish, and rather focus on the very small and very large fish. Even though we did not observe significant differences in the GI microbiota composition when comparing the whole groups of large and small killifish at day 40, we cannot rule out the possibility of differences in microbiota at earlier developmental stages contributing to the differences in size. At sampling all killifish had entered the more developed juvenile stage. Conversely, the differences in size were observed from 20 days post hatch. Human microbiome studies have described an initial variable microbiota heavily influenced by environmental factors (such as mode of delivery, type of feed etc.), which subsequently stabilizes during early development, coinciding with increasing host selection^24^,^25^. A contrary trend has been shown in zebrafish and cod, where there seems to be strong host selection and negligible effects of environmental factors on the microbiota at the larval stage^26^,^27^. If genetic selection factors for gut microbiota develops with age it would seem to be expected that initial differences due to stochastic colonization of larvae will decrease with age in genetically homogenous populations. Reversely, if an initial deterministic (under strong host selection) community composition becomes more influenced by stochastic events as the killifish age, more dissimilar microbiotas would have been expected. The *Artemia* used to feed the killifish were obtained by standardized methods, resulting in similar microbial content in the feed administered to the fish (within Bray Curtis similarity was 0.52). The significant difference found between the *Artemia* microbiota and killifish microbiota also points towards a low influence of the feed microbiota, and strong host selection. Further characterization of the microbiota of early killifish larvae is needed to confirm whether gut microbiota variation is indeed higher in less developed individuals.

### Cod larvae size measurements

**Figure 4:**
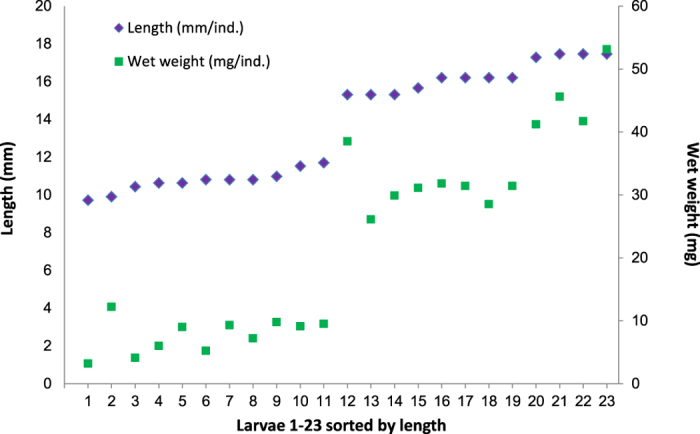
Total length and wet weight of the selected 23 cod larvae at 43 dph.

Twenty-three out of the 30 randomly sampled cod larvae (43 dph) were selected for analysis, excluding misshaped individuals. The larvae were divided in two groups, consisting of 11 small and 12 large larvae, referred to as S1–S11 and L1–L12 respectively. The 23 larvae ranged from 11 to 17 mm in length, and between 3 and 53 mg in weight (Fig. 4). Both average length and weight of the small larvae was significantly lower than for the large larvae (p < 0.005) (for individual measurements see supplementary Table S3).

### Microbiota associated with large and small cod larvae

**Figure 5:**
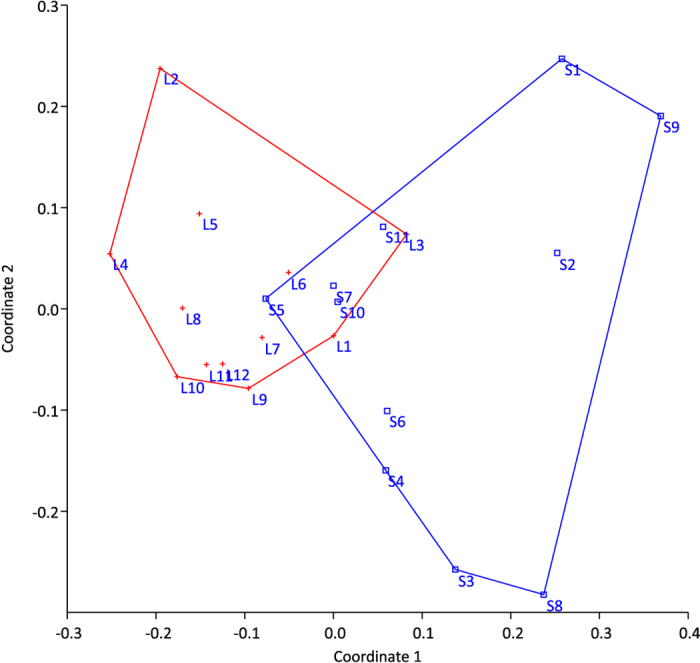
NMDS ordination of microbial community profiles using NMDS with Bray-Curtis similarities for large (L1–L12) and small (S1–S11) cod larvae (stress value < 0.2).

DGGE analysis detected a total of 47 unique bands, of which only one was present in all samples (for gel image see supplementary Fig. S2). Band richness (k) varied between 15 and 26, and the average k values were similar for large and small cod larvae (19.9 and 20.3 respectively). However, both the Shannon diversity index (H′) and Pielou’s evenness index (J′) were significantly different between the two groups (p < 0.005), the small larvae showing greater diversity and evenness than the large larvae. This indicates a more selective process of gut colonization in the large larvae. Recent shifts in niche availability caused by faster growth and maturation of the gut may (also) have led to a less diverse microbiota in larger larvae. The NMDS analysis clearly separates the large and small larvae (Fig. 5), and hypothesis testing with One-Way PERMANOVA confirmed a significant difference in microbiota composition between the two groups (p = 0.0002).

The differences in microbiota between large and small cod larvae cannot be explained by differences in feed or water microbiota. The feed supplied to all the cod larvae in this experiment came from the same batch cultivation system, and larvae were grown in tanks with the same water supply. The role of the feed and water microbiota is therefore assumed to be equal for all larvae, and analysis of feed/water microbiota was thus not performed. In general cod larvae microbiota is established under strong host selection, and tends to be more similar to the microbiota of the rearing water, and the effects of feed microbiota are negligible^27^,^28^.

**Figure 6:**
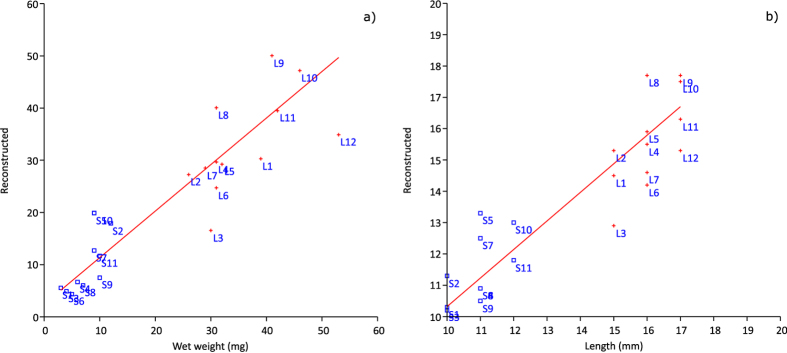
Cabfac reconstruction of size variables of individual larvae (**a**) wet weight (R^2^ = 0.90) and (**b**) total length (R^2^ = 0.89) based on microbiota composition (DGGE).

A Cabfac factor analysis was performed to estimate the effect of cod larvae microbiota composition on larval size (Fig. 6). The Cabfac reconstruction confirms the strong correlation between size and microbiota composition in the cod larvae; 90% of the individual size variation can be explained by differences in microbiota composition across all individuals, and the slope of this relationship was not significantly different from 1 (0.95 and 0.98 for length and weight, respectively).

SIMPER analysis revealed that differences in abundance of 10 bands contributed to more than 70% of the dissimilarities between the small and large larvae. Out of these 10, taxonomy was assigned to 4 (Table 2). The sequenced bands contribute to 34% of the variation between the two samples. The amounts found in large and small larvae were significantly different for all four bands. The most dominating band found in all the large larvae was also present in all but one of the small larvae, but at significantly lower levels. Sequencing revealed that this band most likely represented *Arcobacter* (ɛ-Proteobacteria). Some members of the *Arcobacter* genus are considered foodborne pathogens, but they have also been isolated from oysters and the intestines of healthy livestock, as well as surface and groundwater^29^,^30^,^31^. For the cod larvae in this study this bacterial strain was not correlated with poorer growth, rather on the contrary as intensity of the band was significantly higher in the samples from the large larvae. Simper analysis showed that the difference in abundance of this strain between the large and small larvae contributed to ~18% of the total difference in microbiota composition, with mean abundances of 37.1 *vs* 21.1%. In a recent study by Bakke *et al.*^27^, pyrosequencing of cod larvae at different developmental stages revealed that *Arcobacter* dominated the microbiota of larvae at 17 and 32 dph, constituting 17–77% of the larval microbiota. The high abundance of this bacterium in seemingly healthy larvae in both studies may indicate that this species is a member of the commensal microbiota of cod larvae. Another band with significantly higher abundance in the large larvae was a *Flavobacterium* strain, contributing to 8.3% of the differences in the microbiota, with mean abundances of 10.4% and 3.6% for the large and small larvae, respectively. *Flavobacterium* was also found in larval microbiota at 8 and 32 dph in the study by Bakke *et al.*^27^, at comparable levels.

Two DGGE bands representing *Aliivibrio logei* and *Aliivibrio wodanis* were found to be unique for the small larvae (B39 and B44, Table 2). These two strains are often found in association with known pathogenic strains causing diseases in salmon, but are to our knowledge not connected with any specific disease in cod^32^,^33^. The bands corresponding to these two vibrios were not present in all the small larvae, but were consistently found in the 6 smallest larvae in the “small larvae group”. This may be taken as an indication of an unfavorable colonization that affects larval growth negatively. Similarly, in a culture-based characterization study, fast growing grouper (*Epinephelus coioides*) was found to contain lower levels of vibrios than slow growing grouper^21^.

### Possible role of genetic background on gut microbiota composition

The killifish in this study are expected to have very similar host selection for individuals at the same developmental stage, as they are clonal and therefore genetically homogenous. Any observed variation in microbiota would therefore most likely be due to stochastic events. Stochastic events during the early colonization of fish larvae may lead to establishment of a stable commensal microbiota, or to unstable or detrimental interactions with opportunistic bacteria^34^. Only by excluding the intermediate sized fish from analysis did we find significant differences in the gut microbiota of large and small killifish. A strong genetically encoded host selection may explain why we only observed subtle variation in microbiota composition. As the growth of the large and small killifish started to differ well before metamorphosis into the juvenile stage was complete, it may be that more dissimilar microbiotas initially colonized the larvae. In previous studies however, strong host selection has been indicated at early developmental stages for both cod and zebrafish larvae^26^,^27^. More studies are needed to investigate if there is strong host-selection in the earlier stages of killifish development.

The cod larvae were spawned from mixed parents, and thus have a heterogeneous genetic background. The large differences in microbiota between small and large larvae may be due to differences in host selection factors or subtle differences in development of the larvae, both of which could be influenced by genetic factors. In humans monozygotic twins has been shown to have higher gut microbiota similarity than dizygotic twins^35^, and in mice several quantitative trait loci which control abundances of different taxa have been identified by the use of advanced intercross breeding lines^14^,^16^. Also for other fish species there seems to be higher similarities in microbiota composition in closely related individuals^36^,^37^. Further studies on full sibling groups are needed to investigate whether growth and microbiota composition is more even in a less heterogeneous group of cod larvae.

## Concluding remarks

To conclude, whereas the genetically homogenous killifish only had subtle differences in gut microbiota composition and were significantly different only when comparing the largest and smallest of the sampled fish, the genetically heterogeneous cod larvae had distinct differences in the microbiota of large and small larvae. For both species a strong host selection and a connection between the gut microbiota and the growth of the fish was indicated. Whether the large fish grew better as a result of the different microbiota, or acquired different microbiota as a result of differences in growth, needs further studies. Such studies should have a design which can disentangle the implications of host genetics on selection of gut microbiota. As 16S DGGE has limitations with regards to detecting low abundant species and does not supply information on taxonomy, future studies should use high throughput deep sequencing. The correlation between growth and microbiota is very interesting from a microbial management perspective, where a rewarding concept would be to steer the microbial communities in the rearing water (and thus in the fish gut) towards a microbiota that promotes rapid growth^28^,^38^,^39^.

## Materials and Methods

### Biological material and rearing conditions

All prevailing local, national and international regulations and conventions, and normal scientific ethical practices have been respected during the fish experiments. The cod experiment was approved by the Norwegian Animal Research Authority, and the killifish experiment followed the guidelines of the Animal Care and Use Committee of Nagasaki University, Japan.

### Mangrove killifish

The DAN clonal strain of mangrove killifish was used in this study (obtained from W.P Davies, US Environmental Protection Agency, Florida USA). Larvae from this strain are descendants of one fish collected in Belize, and has been reared for over 10 generations at the Aquaculture Biology lab, Nagasaki University, Japan^23^.

Mangrove killifish eggs were manually dechorionated following the protocol by Koenig and Chasar^40^, using fine forceps to release the larvae. Thirty larvae were reared individually in 100 mL plastic cups, containing 60 mL artificial seawater (Marine Art High, Senju, Saiaku Co., Ltd, Osaka) dissolved to 17 ppt in ozonated tap water. The fish were held at 25 ± 1°C with a photoperiod of 14 h light and 10 h darkness. Water was exchanged every 10 days, coinciding with larval length measurements. Larval length was determined using a digital microscope (Keyence VH-6300). Total length was converted into standard length by the use of the following equations; y = −0.21 + 0.84x. Wet weight was recorded after drying the fish with KimWipes^®^.

Cysts of *Artemia* (Great Salt Lake, Aquafauna Biomarine Inc., CA, USA) were decapsulated with hypochlorite according to the protocol of Sorgeloos *et al.*^41^. Decapsulated *Artemia* (1–2 day old naupli) were added as feed for the killifish, ad libitum every 2–3 days. Rearing water, as well as *Artemia* from 4 different feed “batches” (13, 17, 21 and 27 dph) were sampled for microbiological analysis. When all the fish had reached the juvenile stage at 40 dph, length was measured and the fish were put to death by triacaine methane sulphonate (MS 222). Intestines from all 30 juveniles were sampled for microbiological analysis, using forceps and fine needles.

### Cod larvae

Cod larvae were reared at SINTEF Fisheries and Aquaculture, Trondheim. The cultivation regime included addition of rotifers (fed Reed Mariculture Rotifer diet^®^ and *Pavlova* 1800 paste) from 2 dph, and MarolE (Sintef, Trondheim, Norway) enriched *A. fransiscana* from 18 dph (for details see Supplementary Table S4.). At 43 dph 30 larvae were randomly sampled from identically treated rearing tanks, and put to death using MS 222. Total length of individual larvae was measured by the use of a stereo microscope. Rinsed larvae were dried with KimWipes^®^ before wet weight was determined. Larvae were stored at −80°C before microbiological analysis. As a result of the small size of cod larvae, the guts were not dissected out. As whole cod larvae were used in the microbiota analysis, a contribution from skin-associated microbiota cannot be ruled out. However, the number of skin-associated bacteria have been reported to be low compared to that of the gut microbiota^42^.

### DNA extraction

Killifish intestines and whole cod larvae were homogenized in 200 μl autoclaved seawater using a sterile plastic rod and a syringe. DNA was extracted from the fish, water and *Artemia* samples using the QIAGEN DNeasy blood and tissue kit with some modifications from the manufacturer’s protocol^28^. DNA concentration was measured with a NanoDrop spectrophotometer (Thermo Fisher Scientific).

### PCR-DGGE

To avoid co-amplification of eukaryote DNA, a nested PCR protocol was used to amplify the V3 region of the bacterial 16S rDNA^43^. The quantity and size of PCR products were investigated on 1% agarose gels casted with GelRed^TM^. DGGE was performed as described by Muyzer *et al.*^44^, with the INGENY phorU DGGE system. A denaturing gradient of 35–55% (where 100% corresponds to 7 M urea and 40% formamide) was used in 8% acrylamide gels. Depending on the template concentration, 2.5–15 μl of sample was loaded into the wells, to obtain comparable amounts of PCR product. The gels were run with 0,5 × TAE buffer at 100 V for 17 hours at 60 °C. After electrophoresis, the gels were stained with SYBR Gold (1:10 000 dilution, Invitrogen^TM^) for 1 hour, rinsed with MilliQ water and photographed under UV light (GenBox geldoc system, Syngene).

### Sequence analysis

Selected bands from the DGGE gels were stamped out using a micropipette, and left to dissolve in MilliQ water for 24 hours at 4 °C. After re-amplification as described in Bakke *et al.*^28^, PCR products were purified using QIAquick PCR Purification kit (Qiagen) and DNA sequencing was performed by Eurofins MWG operon using M13R (5′-caggaaacagctatgac-3′) as a sequencing primer. Sequences were then analyzed through the Ribosomal Database Project (RDP) Classifier^45^.

### Gel analysis and statistical analysis

DGGE gel images were analyzed with the Gel2k software^46^ (provided by Svein Norland, Dept. Biology, Univ. Bergen, Norway) to convert band profiles to histograms, resulting in sample-peak area matrices. Individual peak areas for DGGE bands were normalized by dividing by the sum of all peak areas from each respective DGGE profile. The Shannon index (H′)^47^, and the relative diversity, J′ (evenness) were used as measures of diversity in the DGGE profiles, in addition to band richness (k). Student’s t-test (unpaired) was used to investigate significance in differences in Shannon indices, abundance of individual DGGE bands and larval growth measurements. One-way PERMANOVA (non-parametric MANOVA^48^) based on Bray-Curtis similarity was used to test for differences in DGGE profiles between groups of samples. Ordination by Non-metric multidimensional scaling (NMDS) based on Bray-Curtis similarity was used to visualize the similarity/dissimilarity in bacterial community profiles among the samples^49^. SIMPER analysis was performed to identify which DGGE bands contributed most to dissimilarities between samples. CABFAC analysis (a factor analysis of abundance data with associated environmental data) was used to reconstruct larval weight/length based on microbial community DGGE profiles). The multivariate analyses were performed with the PAST software package^50^.

## Additional Information

**How to cite this article**: Forberg, T. *et al.* Correlation between microbiota and growth in Mangrove Killifish (*Kryptolebias marmoratus*) and Atlantic cod (*Gadus morhua*). *Sci. Rep.* **6**, 21192; doi: 10.1038/srep21192 (2016).

## Supplementary Material

Supplementary Information

## Figures and Tables

**Table 1:** Sequenced bands (identified through RDP) from the killifish DGGE-gel, with average dissimilarities based on SIMPER analysis with Bray-Curtis similarity.

Band #	Average dissimilarity	% Mean abundance large killifish	% Mean abundance small killifish	% Contribution dissimilarity	T test p value	Class/Genus seqmatch RDP
B38	3.21	8.19	3.68	6.9	0.207	*Vibrio*
B36	3.04	7.42	1.97	6.5	0.141	*Vibrio*
B45	3.07	7.14	11.8	6.6	0.069	*Microbacteriaceae*
B6	1.91	3.63	5.74	4.1	0.245	*Staphylococcus*
B42	1.07	2.66	3.86	2.3	0.232	*Microbacteriaceae*
B39	0.61	2.72	2.37	1.3	0.598	*ϒ-Proteobacteria*

**Table 2:** Sequenced bands (identity from RDP) from the cod larvae DGGE-gel, with average dissimilarities based on SIMPER analysis with Bray-Curtis similarity.

Band #	Average dissimilarity	Mean % abundance large cod	Mean % abundance small cod	% contribution to dissimilarity	T test p value	Class/Genus Seqmatch RDP
B18	4.371	10.4	3.62	8.3	0.024	*Flavobacteria/Polaribacter*
B26	9.472	37.1	21.1	17.9	0.003	*Arcobacter*
B39	2.005	0	4.01	3.8	0.015	*Aliivibrio*
B44	1.859	0.0417	3.72	3.5	0.027	*Allivibrio*
