# Supplementary material for: Correlation between microbiota and growth in Mangrove Killifish (Kryptolebias marmoratus) and Atlantic cod (Gadus morhua)
Source: Sci Rep. 2016 Feb 15;6:21192. doi: 10.1038/srep21192 (PMC4753419; doi:10.1038/srep21192)
Supplement: Supplementary Information [file srep21192-s1.pdf]

Correlation between microbiota and growth in Mangrove Killifish (*Kryptolebias marmoratus*) and Atlantic cod (*Gadus morhua*)

Torunn Forberg[1], Eli Bjørnø Sjulstad[1], Ingrid Bakke[1], Yngvar Olsen[2], Atsushi Hagiwara [3], Yoshitaka Sakakura[3] and Olav Vadstein*

Norwegian University of Science and Technology, [1]Department of Biotechnology and [2]Department of Biology, N7491 Trondheim, Norway

Nagasaki University, Graduate School of Fisheries and Environmental Sciences[3], 1-14 Bunkyo-machi Nagasaki 852-8521, Japan

*Olav.vadstein@ntnu.no

SUPPLEMETARY MATERIAL

Supplementary Table S1: individual measurements of killifish juveniles 40 dph.

Individual fish	Length (cm/ind.)	Wet weight (g/ind.)
S1	10,12	0,0181
S2	10,28	0,0184
S3	10,33	0,0232
S4	10,38	0,0246
S5	10,41	0,0240
S6	10,48	0,0228
S7	10,49	0,0210
L1	11,50	0,0282
L2	11,51	0,0254
L3	11,54	0,0286
L4	11,67	0,0316
L5	11,84	0,0318
L6	11,84	0,0326
L7	12,04	0,0296

Supplementary Table S2: Average Bray Curtis Similarities (in bold text) for large and small killifish, water and *Artemia* samples, with standard deviations.

Artemia (within)	Artemia vs. Water	Large killifish (within)	Large killifish vs. Water	Large killifish vs. Artemia	Small killifish (within)	Small killifish vs. Water	Small killifish vs. Artemia	Small killifish vs. Large killifish
**0,52**	**0,43**	**0,46**	**0,30**	**0,29**	**0,64**	**0,36**	**0,35**	**0,53**
0,04	0,02	0,04	0,02	0,02	0,01	0,02	0,01	0,02

Supplementary Table S3: individual measurements of cod larvae 43 dph

Individual larvae	Length (cm/ind.)	Wet weight (mg/ind.)
S1	1,0	0,003
S2	1,0	0,012
S3	1,0	0,004
S4	1,1	0,006
S5	1,1	0,009
S6	1,1	0,005
S7	1,1	0,009
S8	1,1	0,007
S9	1,1	0,010
S10	1,2	0,009
S11	1,2	0,010
L1	1,5	0,039
L2	1,5	0,026
L3	1,5	0,030
L4	1,6	0,031
L5	1,6	0,032
L6	1,6	0,031
L7	1,6	0,029
L8	1,6	0,031
L9	1,7	0,041
L10	1,7	0,046
L11	1,7	0,042
L12	1,7	0,053

Supplementary Table S4: Feeding schedule for the sampled cod larvae as a function of days after hatching (DPH).

DPH	Feed	Producer of feed
1-20	Green water (Nannochloropsis sp. paste) Reed Mariculture	Green water (Nannochloropsis sp. paste) Reed Mariculture
2-22	Rotifers (Brachionus plicatilits „Nevada’) with a cultivation diet of rotifer diet and Pavlova paste SINTEF Fisheries and Aquaculture (Rotifers), Reed Mariculture (Pavlova paste)	Rotifers (Brachionus plicatilits „Nevada’) with a cultivation diet of rotifer diet and Pavlova paste SINTEF Fisheries and Aquaculture (Rotifers), Reed Mariculture (Pavlova paste)
18-43	A. franciscana enriched with MarolE SINTEF Fisheries and Aquaculture	A. franciscana enriched with MarolE SINTEF Fisheries and Aquaculture

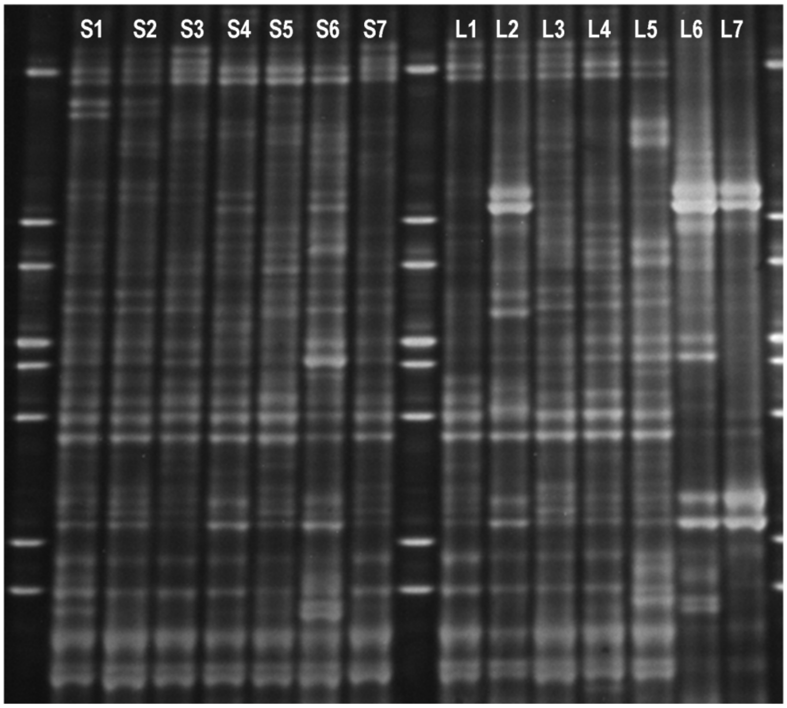

Supplementary Figure S1: DGGE gel DAN killifish samples S1-S7 and L1-L7.

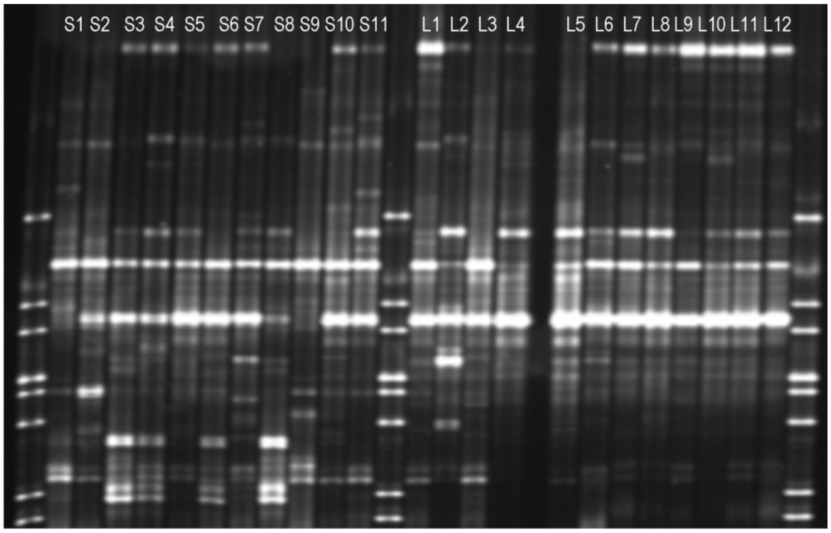

Supplementary Figure S2: DGGE gel cod larvae samples, S1-S11 and L1-L12.
